# Li-Pos: A Light Positioning Framework Leveraging OFDM for Visible Light Communication

**DOI:** 10.3390/s21134310

**Published:** 2021-06-24

**Authors:** Jianbin Wu, Sami Ahmed Haider, Muhammad Irshad, Jehangir Arshad, Sohail M. Noman, Aparna Murthy

**Affiliations:** 1 Department of Computer Science and Engineering, Zhejiang Normal University, Jinhua 321004, China; wjb@zjnu.cn; 2 Department of Computing, University of Worcester, Henwick Grove WR26AJ, UK; s.ahmedhaider@worc.ac.uk; 3 Department of Electronics and Information Engineering, The Hongkong Polytechnic University, Hung Hom, Hong Kong, China; 4 Department of Electrical and Computer Engineering, Comsat University Lahore, Punjab 54000, Pakistan; jehangirarshad@cuilahore.edu.pk; 5 Department Cell Biology and Genetics, Shatou University Medical College, Shantou 515000, China; mn.sohail@stumail.ysu.edu.cn; 6 EIT, PEO, Toronto, ON M4B 1B3, Canada; aparnasm11@gmail.com

**Keywords:** visible light communication, full-width half maximum, positioning, power and delay spread, optical orthogonal frequency division multiplexing

## Abstract

The design of solid-state lighting is vital, as numerous metrics are involved in their exact positioning, and as it is utilized in various processes, ranging from intelligent buildings to the internet of things (IoT). This work aims to determine the power and delay spread from the light source to the receiver plane. The positions of the light source and receiver were used for power estimation. We focus on analog orthogonal frequency-division multiplexing (OFDM) in visible light communication (VLC) and assess the area under the curve (AUC). The proposed system was designed using modulation techniques (i.e., quadrature amplitude modulation; QAM) for visible light communication (VLC) and pulse-width modulation (PWM) for dimming sources. For the positioning and spreading of brightness, the proof-of-concept was weighted equally over the entire area. Therefore, the receiver plane was analyzed, in order to measure the power of each light-emitting diode (LED) in a given area, using the delayed mean square error (MSE). A framework was applied for the placement of LEDs, using full-width at half-maximum (FWHM) parameters with varying distances. Then, the received power was confirmed. The results show that the AUC using DRMS values for LEDs significantly increased (by 30%) when the number of source LEDs was changed from four to three. These results confirm that our system, associated with the simple linear lateration estimator, can achieve better energy consumption.

## 1. Introduction

Light is a vital resource that plays multifaceted roles, including illumination and energy. In recent years, much research has been performed on visible light communication (VLC) technology, which has received increasing investment and has been used in a growing number of applications in the communications industry [1,2]. As most current visible indoor communication systems suffer from prolonged time delays, low position accuracy, and high power consumption, VLC is an appealing solution for bringing wireless communication to regions where radio frequency (RF) communications are rarely used, such as hospitals, oil or chemical plants, and aircraft [3,4,5].

Conversely, the localization process can be realized by visible light positioning (VLP) systems using visible light signals. Position estimation can be achieved by using photodiode-based VLC receivers, utilizing the signals emitted by an LED transmitter at a known location [6,7,8]. VLP systems based on VLC may also be equipped with a variety of multiplexing schemes, including time-division multiplexing (TDM) and space-division multiplexing (SDM) [9,10].

In general, indoor communication distortion is caused by the propagation of optical signals with multi-directional distortion. Orthogonal Frequency-Division Multiplexing (ODDM) is an effective technique to combat the inter-signal interference caused by multi-path reflection in VLC systems [11,12]. Unlike radiofrequency OFDM systems, orthogonal modulation in light intensity modulation and direction detection (IM/DD) systems are very complicated [13,14]. To improve the optical efficiency, researchers have studied several OFDM-based VLC systems, such as asymmetrically clipped optical orthogonal frequency-division multiplexing (ACO-OFDM), DC-biased optical orthogonal frequency-division multiplexing (DCO-OFDM), and PAM-modulated discrete multi-tones (PAM-DMT) [15].

Further, the location problem has generally been considered to be non-cooperative, and only position measurements are performed between the target point and the reference point [16]. The main principle behind the cooperation-based approach is to use measurements between target nodes (located) and reference nodes. In this way, the positioning accuracy can be improved [17,18,19]. Received signal strength (RSS) and received light intensity (RLI) algorithms are the most commonly used algorithms for determining indoor positions. In [20,21], the authors used a unipolar scheme to achieve Hermitian symmetry. In [22], power attenuation was used to estimate receiver coordinates using on–off keying (OOK). Two amplitude shift keying constant envelope (2ASK-CE)-OFDM coding has been implemented for positioning in a real-time VLC environment [23]. The complexity of demodulation was addressed using forward error correction (FEC) technology. In addition, least squares estimation was used in combination with time-division multiplexing (TDM) to improve the positioning accuracy. The practicability of orthogonal frequency-division multiple access (OFDMA) with visible light communication has been studied by [24,25]; however, the system throughput was degraded, due to the selection of sub-carriers.

When evaluating the performance of a VLC system, one of the most important factors to consider is the size of the test area. The larger the test platform, the more practical the solution. Table 1 compares the performance of current photodiode (PD)-based solution systems. In addition to the test area size, parameters such as the positioning speed, size of the receiving device, cost, power consumption, and complexity also determine the robustness of a system.

In this paper, we propose a received signal strength-based least squares lateration algorithm. The lateration algorithm increases the accuracy of location estimation by using redundancy in the number of access points and applying a least-squares approximations to the received signal strength values. The LED characteristics are calculated to validate the proposed positioning model. Visible light positioning and visible light communication are realized simultaneously in the same frequency band as that of OFDM. The power and delay are measured at the receiver plane. The sub-carrier block is divided between multiple LEDs, which helps calculate the distance between the transmitter and receiver to improve the positioning accuracy. Moreover, a random data sequence with constant average power is generated as a position signal that also contains data information. The known coordinates of the LED transmitter result in unknown coordinates of the receiver (photodetector). The proposed positioning algorithm is based on an RSS lateration technique, and the results of our experiments validate the accuracy of the algorithm, even in disperse optical channels.

The rest of the paper is structured into five sections, as follows: Section 2 provides the system model, including the principles of VLC, OFDM simulation, and channel estimation. Section 3 presents the experimental setting and results. Finally, our conclusions are summarized in Section 4.

## 2. System Configuration Methodology

### 2.1. VLC Principle and System Model

The proposed system comprised a typical room with dimensions of 6m×6m×4m, where four highly reliable LED sources were placed at a height of 2.15m.

**Figure 1:**
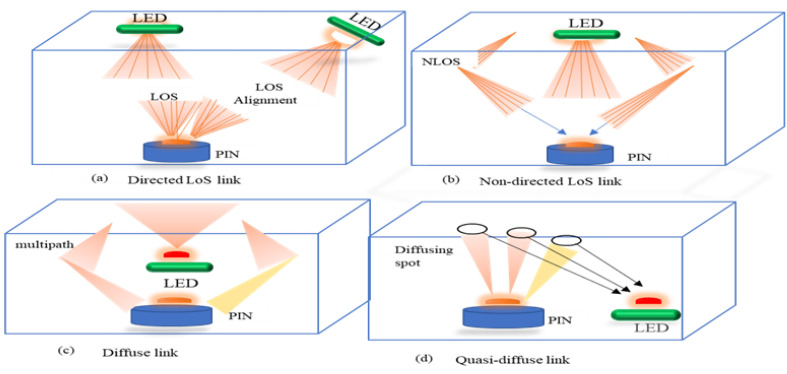
Typical Optical Link Diagram.

Data modulation was performed by the driver circuit before it was transmitted by the LEDs. The PD receivers were placed at a height of 0.85m, with a field-of-view (FoV) of $30^{\circ }$ and a receiving area of $100\;{\mathrm{mm}}^{2}$. The decentralized nature of VLC channels makes orthogonal frequency-division multiplexing an attractive modulation technique for indoor position estimation. In addition, both LEDs and PDs provide low-cost solutions for optical carriers and can be applied with intensity modulation with direct detection. The PD measures the light power emitted from the LED in the field-of-view and performs positioning based on the power measurement result. The OFDM-based system is used in order to mitigate the effects of multi-path distortion due to the optical channel. Moreover, the transmitted waveform is modulated into instantaneous power, proportional to the current at the receiving end, and there is no need for frequency and phase information. Furthermore, the relationship between the LED’s optical power and current is considered the turn-on voltage (TOV), which is the threshold for the device in an off or on state. Figure 1 shows a typical optical link diagram of the visible light with coded information.

### 2.2. Motivation and Observation

**Figure 2:**
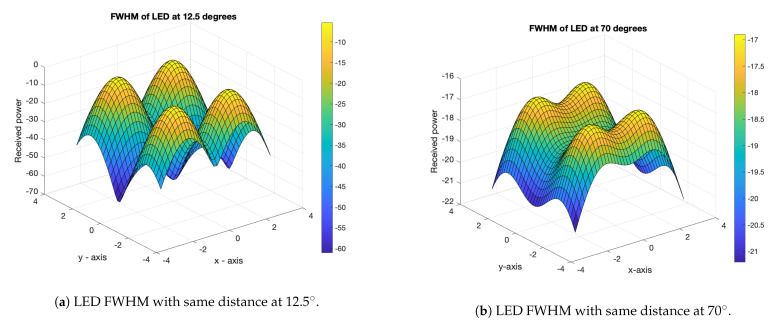
FWHM at two different angles.

The main objective of this research was to evaluate the time-dispersion efficiency of LEDs at different full-width half-maximum (FWHM) values for indoor VLC systems. A simple set of quadratic and linear equations were solved, in order to produce an accurate two-dimensional position estimate, which depends on the DRMS with a given FWHM intensity. We applied an analytical lower bound for the minimum optical SNR. We also determined that the deploying power of the LED influences the BER performance for the receiver location. We observed that a shift in the obtained power through the equidistant LED provides energy efficiency and avoids ISI for positioning demonstration, where the LEDs exclusively occupy sub-carrier blocks. Thus, given the location of the receiver, each LED has the power threshold to initiate communication. The positioning error was calculated using OFDM with N=4 LEDs; then, we reduced the number of LEDs that were placed in the ceiling. This approach offers a reasonable degree of freedom to achieve interesting lighting effects, in terms of light positioning and luminous flux distribution in homes and in workplaces. This research addresses the lighting position between two LEDs having full-width half-maximum values of $12.5^{\circ }$ and $70^{\circ }$, respectively, with a fixed height from the photodiode. This was achieved by maintaining the same full-width half-maximum (FWHM) distance between scenarios, as shown in Figure 2.

Figure 2 was used to determine the FWHM pulse width with an error-free transmission, through the use of forwarding error correction (FEC) for all LEDs. Intensity modulation and direct detection are commonly used with VLC; however, we used the pulse-width modulation (PWM) technique to modulate the intensity of the signal which is integrated into the OFDM signal during transmission and reception. Previous researchers have suggested diverse optical-based OFDM techniques. For the sake of brevity, we considered the DC-biasing OFDM signal. The clipped bipolar OFDM signal is given by [30]:

$$x_{c}\left(t\right)=\left\{\begin{array}{cc} x(t), & \;\mathrm{if}\;x(t)>0\\ 0, & \;\mathrm{otherwise}, \end{array}\right.$$

where x(t) is the unclipped signal.

Moreover, the DC optical (DCO)-OFDM is made unipolar by adding the DC bias [1], which is given by (1):

(1) $$x\left(t\right)=x_{0}\left(t\right)+\beta_{DC}+N,$$

where $x_{0}\left(t\right)$ is the original vector signal, $\beta_{DC}$ is the added DC bias (which varies with the QAM constellation), and *N* is the noise component that is added to the DC signal. The DC bias value is not fixed for all constellations. We used pulse-width modulation (PWM) to dim the LEDs.

(2) $$y\left(t\right)=x\left(t\right)*P_{\mathrm{pwm}}\left(t\right),$$

where y(t) is the signal driving the LED, x(t) is the input, and $P_{\mathrm{PWM}}\left(t\right)$ is the PWM signal, with a pulse duration equal to that of the OFDM pulse [1,31]. In the ACO-OFDM system, the data transmission is performed in the odd sub-carriers in the Hermitian form, represented by:

$$[0,x_{1},0,x_{3},0,x_{5},\cdots ].$$

The Hermitian form of the signal is also a vector quantity representing the signal to be transmitted. The pulse duration is given by (3). Here, *R* is the ratio of OFDM pulse duration to the total time period of PWM [20].

(3) $$T_{ACO}=\frac{2\frac{N}{4}\log_{2}M}{R(N+N_{CP})}.$$

The transmission model of DCO-OFDM, with *N* sub-carriers having a pulse duration of *T* for an *M*-ary QAM, is given in (4):

(4) $$T_{DCO}=\frac{2\left(\frac{N}{2}-1\right)\log_{2}M}{R(N+N_{CP})},$$

where $N_{CP}$ is the OFDM’s signal prefix cycle length.

In the optical wireless channel setting, the OFDM system model is based on IM/DD technology. The signal x(t) is a real, unipolar signal that uses a PWM dimming LED and is given by Equation (2). The DCO-OFDM uses both even and odd sub-carriers but, due to Hermitian symmetry, half of the sub-carrier band is not used [20]. Equation (3) yields the spectral efficiency of the QAM signal. Due to the application of ACO-OFDM in odd-numbered sub-carriers, this technique is not very effective.

The input data are transformed into the Hermitian symmetry form. For an integer-based square matrix, there is a unimodular matrix. such that H=A∗U, where A is the unimodular real matrix with a determinant of ±1. The Hermitian normal form of a non-singular matrix A provides a non-singular integer square matrix H, given by [32]:

(5) $$H_{jj}\geq 0;\;\;H_{jj}\leq H_{ij}\leq \frac{H_{jj}}{2}\;\mathrm{for}\;j>i.$$

If A is a matrix of polynomials, then U is a constant; otherwise, it is an upper-triangular matrix of A. Using a 64-QAM modulation technique, we transform the signal to the time domain.

### 2.3. Channel Estimation

According to [33], the channel noise is modeled as an additive white Gaussian noise (AWGN), and $P_{t}$ is the instantaneous optical power.

(6) $$I_{p}\left(t\right)=P_{t}\left(t\right)⊗h\left(t\right)+n\left(t\right),$$

where $P_{t}\left(t\right)=\lim_{t\to \infty }\frac{1}{T}\int_{0}^{T}P_{i}\left(t\right),\;P_{i}\left(t\right)>0$, h(t) is the impulse response of the channel, and n(t) is white Gaussian noise.

**Figure 3:**
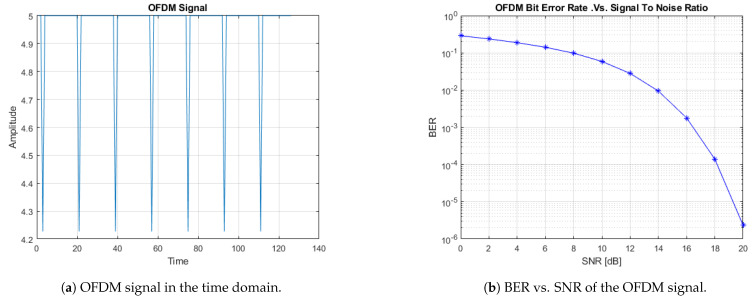
OFDM signal simulation.

The PWM-OFDM characteristic in channel estimation is simpler than switching or dimming the LED circuit. As can be seen in Figure 3a, the generic peak-to-average power ratio (PAPR) does not cause hindrance, as the signal polarity is consistent. Figure 3b illustrates the variation in the typical SNR (signal-to-noise ratio) in dB, compared to the BER of the OFDM signal. As we considered an AWGN channel for 64-QAM with an SNR varying between 0 and 25 dB, we obtained the BER as a waterfall diagram. As the SNR increased, the BER seemed to decrease linearly between 10 and 18 dB.

The signal emitted by the LED was encoded with the assigned sub-carrier. In the proposed method, four LEDs are used. The extended fast Fourier transform (XFFT) was assumed as the size of the FFT [34] in the signal. The sub-carriers were arranged in the form of a block, where each block consisted of eight sub-carriers; that is, a 1/8 XFFT sub-carrier. Each LED contained two sub-carrier blocks. The actual output of optical orthogonal frequency-division multiplexing employed Hermitian symmetry.

Each LED, containing a sub-carrier block, is presented in Figure 3a. The fast Fourier transform was carried out on each LED. The LED output was in the form of a light signal, which was transmitted to the photodiode (PD) through the indoor channel, and the PD compiled light concurrently from all LEDs with various transmission delays.

**Figure 4:**
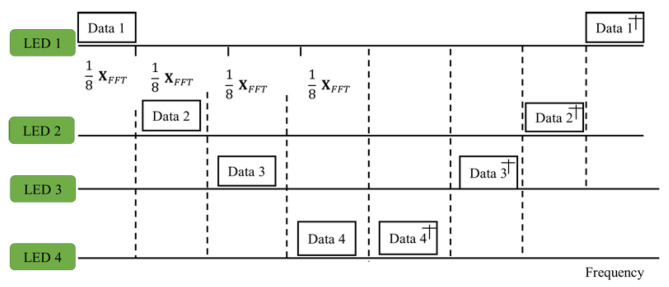
OFDM for VLP Hermitian symmetry.

The signal sensed by the target LED needs to be properly integrated at the receiver. After the fast Fourier transform (FFT), the full bandwidth of the frequency domain signal was obtained at the PD. The average power of each sub-carrier block is shown in Figure 4.

**Figure 5:**
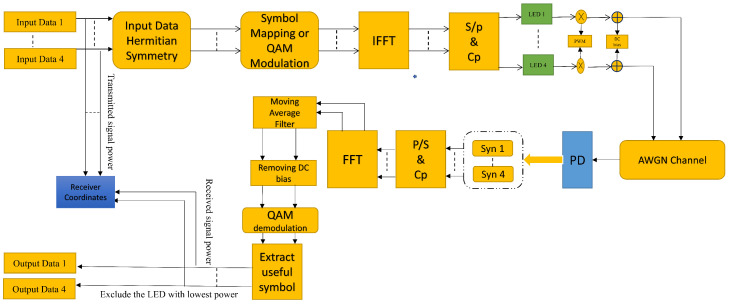
OFDM transmitter and receiver configuration for both communication and positioning purposes.

By evaluating the average power, the distance between the transmitter and receiver can be calculated for each pair of sub-carrier blocks. The longer the transmission distance, the weaker the signal energy and the larger the noise. When using such a long transmission distance with least squares estimation, positioning errors may occur. The block diagram of OFDM, including visible light communication and positioning, is shown in Figure 5.

This block diagram represents the overall functionality of the system. In the system, we ensured that the input data has Hermitian symmetry, then performed symbol mapping and QAM modulation. After modulation, the main building blocks of OFDM, such as serial-to-parallel (S/P) conversion and cyclic prefix, are implemented. The given signal is then modulated using the PWM technique and DC bias is added to the signal. At the receiver, the signal was decoded and converted to the frequency domain; a moving average filter was used to recover the signal, which was then applied to the QAM demodulator.

### 2.4. Distance Estimation

The authors in [35] proposed calculating the distance $d_{i}$ between the *i*-th transmitter and *j*-th receiver at a transmitted power $P_{t}$, as described in (7):

(7) $$d_{i}=\sqrt[{4}]{\frac{AT_{s}\left(\psi \right)g\left(\psi \right)P_{t}{\left(H-h\right)}^{2}}{\pi P_{r}\left(i\right)}},$$

where (H−h) is the vertical distance between the transmitter and receiver, $T_{s}\left(\psi \right)$ is the transmitter function of the optical filter, and g(ψ) is parabolic concentrator gain.

Assume that LED-1 is located at the greatest distance. Then, to improve the positioning accuracy, it can be excluded. Then, a set of four quadratic equations can be formed, according to the lateration algorithm given by (8):

(8) $$\left\{\begin{array}{c} {\left(x-x_{1}\right)}^{2}+{\left(y-y_{1}\right)}^{2}=d_{1}^{2},\\ {\left(x-x_{2}\right)}^{2}+{\left(y-y_{2}\right)}^{2}=d_{2}^{2},\\ {\left(x-x_{3}\right)}^{2}+{\left(y-y_{3}\right)}^{2}=d_{3}^{2},\\ {\left(x-x_{4}\right)}^{2}+{\left(y-y_{4}\right)}^{2}=d_{4}^{2}. \end{array}\right\}$$

The exclusion of LED-1, with the longest distance and lowest average power, is given by (9):

(9) $$\left\{\begin{array}{c} {\left(x-x_{2}\right)}^{2}+{\left(y-y_{2}\right)}^{2}=d_{2}^{2},\\ {\left(x-x_{3}\right)}^{2}+{\left(y-y_{3}\right)}^{2}=d_{3}^{2},\\ {\left(x-x_{4}\right)}^{2}+{\left(y-y_{4}\right)}^{2}=d_{4}^{2}. \end{array}\right\}$$

The exclusion of another LED, LED-3, with the next longest distance and lowest average power, is defined by:

(10) $$\left\{\begin{array}{c} {\left(x-x_{3}\right)}^{2}+{\left(y-y_{3}\right)}^{2}=d_{3}^{2},\\ {\left(x-x_{4}\right)}^{2}+{\left(y-y_{4}\right)}^{2}=d_{4}^{2}, \end{array}\right\}$$

Generalizing the above equations, the linear equation is obtained such that Ax=b, where

(11) $$A=\left[\begin{array}{cc} 2(x_{m}-x_{1}) & 2(y_{m}-y_{1})\\ 2(x_{m}-x_{2}) & 2(y_{m}-y_{2})\\ \vdots & \vdots \\ 2(x_{m}-x_{m-1}) & 2(y_{m}-y_{m-1}) \end{array}\right],$$

(12) $$B=\left[\begin{array}{c} x_{1}^{2}-x_{m}^{2}+y_{1}^{2}-y_{m}^{2}+d_{1}^{2}-d_{m}^{2}\\ x_{2}^{2}-x_{m}^{2}+y_{2}^{2}-y_{m}^{2}+d_{2}^{2}-d_{m}^{2}\\ \vdots \\ x_{m-1}^{2}-x_{m}^{2}+y_{m-1}^{2}-y_{m}^{2}+d_{m-1}^{2}-d_{m}^{2} \end{array}\right],$$

(13) $$X={\left(A^{T}A\right)}^{-1}A^{T}B.$$

In the least-squares estimation, in order to identify the three-dimensional receiver position, different combinations of LEDs are simulated. The matrix representation for calculating the least-squares estimation is given by Equations (8)–(11).

## 3. Result and Discussion

In this section, the simulation results are reported, in order to investigate the performance of the proposed system. We considered a VLP scenario-based indoor multi-path optical channel, where four LEDs (N=4) were used as transmitters in a room. The orientation and location of the LED transmitters and PD receivers are provided in Table 2 [23,36].

The system is over-determined, as the number of LEDs is greater than required, in terms of illumination and positioning in the specified area. Thus, in order to achieve the optimal value, we reduced the number of LED lights and analyzed the DRMS with three and two LEDs. We simulated four identical LEDs mounted in the ceiling; the LEDs were distributed evenly, and the receivers were parallel to all LEDs and placed at a specific height. The characteristics of the LEDs are given in watts, with viewing angles of $12.5^{\circ }$ and $70^{\circ }$. The illumination level ranged from 100 lux to 500 lux, thus satisfying the ISO requirements for lighting in workrooms. The transmitter parameters used in the simulation were taken from the datasheet of the commercial product CREE XLamp^®^ XT-E [37]. The elevation and azimuth angle given is for the LEDs comprising the transmitter plane. The luminous flux was detected by a receiver placed at a height of 0.85 m from the floor. The LEDs were assumed to operate in their linear region with a current rating not exceeding 1 A, and the switching frequency was about 500 kHz. We assumed an OFDM system with 64 sub-carriers, including 32 data transmission sub-carriers, all synchronized in a block with a data rate of 0.44 Mb/s.

MATLAB simulation was performed on a 3.30 GHz/8 GB RAM non-dedicated Windows machine, with a baseband signal of 1 M baud (1 MS/s) [23,36]. To generate the real values, we applied the functional analysis operator; hence, all points of the spectrum were real numbers, regardless of whether the input data were Hermitian [38]. The next step was to map the symbols and compute the inverse fast Fourier transform (IFFT). Pulse-width modulation (PWM) was applied to the digitized waveform; then, the level was shifted by adding a DC bias value. On the receiver side, data were extracted by performing the FFT algorithm. A moving average filter was used to smooth the data with a pole located at (1,−0.55). The transfer function was applied to filter the incoming data stream. A moving average filter with length *N* is given by:

(14) $$y\left(n\right)=\frac{1}{N}\sum_{i=-N+1}^{0}x\left(n+i\right).$$

**Figure 6:**
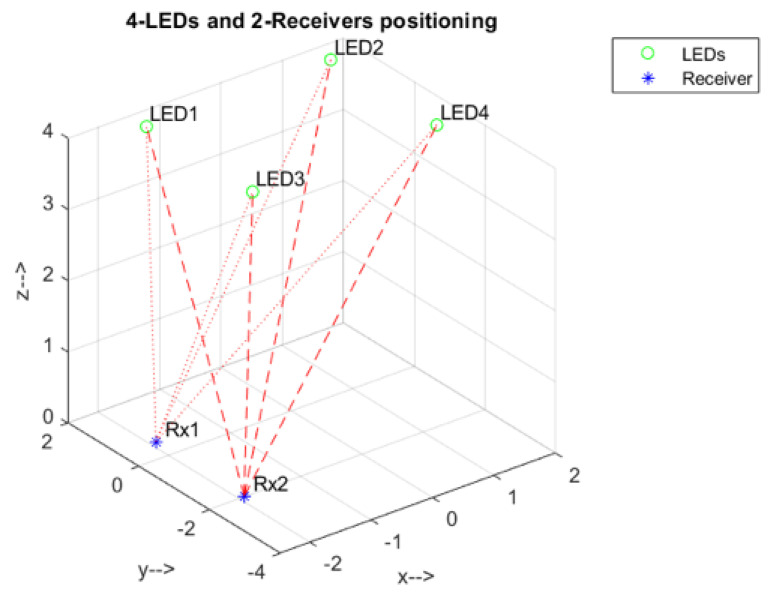
Spatial distribution of LEDs and receivers.

The output of a moving average filter is a statistical vector quantity. The current value is the average of *N* previous samples. The output results in the convolution of input samples with a rectangular pulse of length *N* and height $\frac{1}{N}$. The procedure was verified by investigating the delay root mean square at the room diagonal, where the receivers were located at the coordinates (−2.5,−0.5,0.5) and (−2.5,−3,0.5). The relative positions of LEDs and receivers are shown in Figure 6.

**Figure 7:**
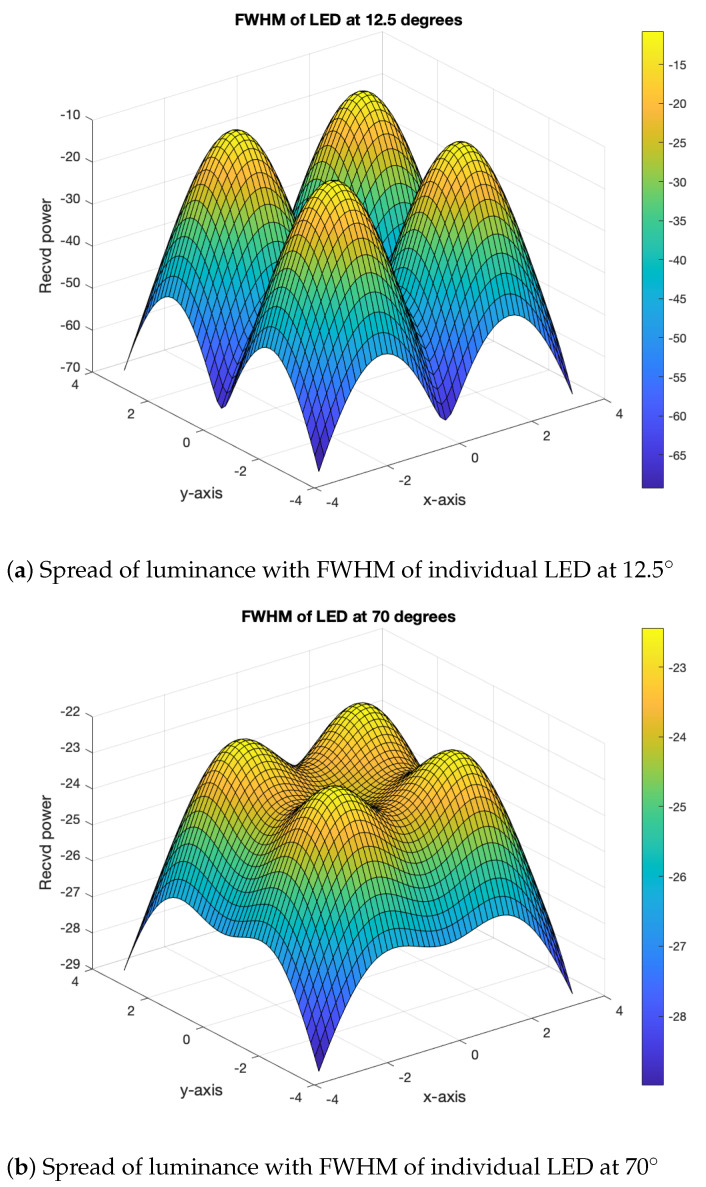
Distance-specific spread of luminance with FWHM.

It can be observed that excluding the LED with the lowest received signal enhanced the positioning accuracy, as verified by the simulation results shown in Figure 7a,b.

With off-the-shelf LEDs and full-width at half-maximum (FWHM) brightness ranges from $12.5^{\circ }$ to $70^{\circ }$, as shown in Figure 7a,b, a narrower FWHM led to discrete peaks, while a wider FWHM produced overlapping peak [39]. The total power is a sum of the individual radiometric values of the LED, making the LED a highly directional source. With an additional optical source, the beam can be widened; even adjusting the position of the device can provide a wider beam.

**Figure 8:**
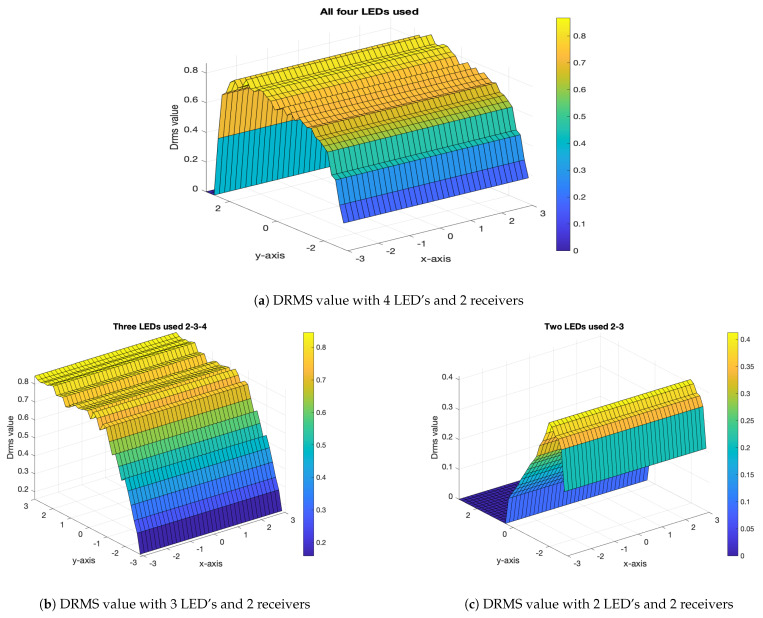
DRMS delay at a different number of LED’s.

Figure 8a shows the DRMS expansion of four LEDs and the spatial distribution of the receiver. Figure 8b,c shows the Lambertian distribution of the LEDs when the number of LEDs was reduced to three and two, by removing the first and third LEDs, respectively, thus showing the spatial distribution of LEDs. The solid angle of the source was received at the receiver plane.

The selection of the LEDs was based on empirical redistribution, such as removing LED-2 or LED-3 and reducing the number of LEDs to three, which showed a significant impact on RMS delay expansion. Removing one LED while retaining the others resulted in an increase in the DRMS value. The LEDs chosen for the simulations had two FWHM ranges (i.e., $12.5^{\circ }$ and $70^{\circ }$), which define the solid angle as light propagates in the given area. Based on these values, the DRMS was simulated for the two scenarios. In the two scenarios, the four LEDs formed a grid to illuminate the given room or area. Once the DRMS was obtained, the area under the curve (AUC) was computed as given by Table 3. The AUC is a single scalar value that provides a metric for comparing the positioning accuracy of the LEDs by varying the number of LEDs. The simulation results in Figure 8 show the AUCs when varying the LEDs, as well as the effect on the coverage area. The theoretical explanation is given in Appendix A.

## 4. Conclusions

In this work, we focused on analog orthogonal frequency-division multiplexing (OFDM) in visible light communications (VLC) and analyzed the area under the curve (AUC). The proposed system uses QAM modulation in addition to OFDM for visible light communication and pulse-width modulation for dimming sources. The receiver plane was analyzed, in order to measure the power of each light-emitting diode in a given area with delayed mean square error. The proposed system provides a framework for positioning LEDs by using full-width at half-maximum (FWHM) parameters with varying distances and comparing the received power. The results show that the AUC based on the DRMS value for LEDs increased significantly (by 30%) when changing the number of LED sources from four to three. These results confirmed that our system, associated with the simple linear lateration estimator method, can improve the associated energy consumption.

## Figures and Tables

**Table 1:** Analysis of PD-based indoor VLC systems.

System	Positioning Algorithm	Test Bed Size	LED Parameters
[9]	RSS triangulation	6m×4m×4m	4 LEDs, 16 W TDM
[26]	RSS finger printing	4m×4m×3m	16 LEDs
[27]	RSS modeling	20m×20m×3m	9 LEDs, TDM
[28]	TDOA trilateration	5m×5m×3m	3 LEDs, FDM
[29]	AOA triangulation	6m×6m×4m	4 LEDs, TDM
Proposed work	RSS lateration	6m×6m×4m	4 LEDs, OFDM

**Table 2:** Simulation parameters.

Parameters	Values
Room Size	6 m × 6 m × 4 m
Reflectance of ceiling	0.35
Reflectance of wall	0.8
Reflectance of floor	0.60
Coordinates of LED Source	(−1.5,−1.5,4),(−1.5,1.5,4)
	(1.5,−1.5,4),(1.5,1.5,4)
Transmitter power from the LED	15 W
Half-width at half-maximum	$12.5^{\circ }$ and $70^{\circ }$
Center luminous flux	300 lm
Elevation of the LED source	$-90^{\circ }$
The azimuth of LED source	$0^{\circ }$
Height of LED source	2.15 m
Detection physical area of the receiver	100 mm${}^{2}$
Height of receiver	0.85 m
Field-of-View of receiver	$70^{\circ }$
Azimuth of receiver	$0^{\circ }$
Elevation of receiver	$90^{\circ }$
Spacing between receiver position	2.5 m
Coordinates of first receiver	(−2.5, −0.5, 0.5)
Coordinates of second receiver	(−2.5, −3, 0.5)

**Table 3:** Positioning matrix and DRMS values using AUC.

Number of LEDs	Positioning	AUC (ins)
4	(−1.5,−1,5,4),(1.5,1.5,4),(1.5,−1.5,4),(−1.5,1.5,4)	1.2573
3	(−1.5,−1.5,4),(1.5,1.5,4),(−1.5,1.5,4)	1.7951
2	(1.5,1.5,4)(−1.5,1.5,4)	1.378

## Data Availability

Not applicable.

## Appendix A

### The Proof of Proposition 1

The root-mean-square delay spread is a measure of multi-path-induced inter-symbol interference. It provides an estimate for the normalized delay due to multiple reflections.

$$\mu =\frac{\sum_{i=1}^{M}P_{d,i}t_{d,i}+\sum_{j=1}^{N}P_{ref,j}t_{ref,j}}{P_{r}},$$

where $P_{d,i}$ and $P_{ref,j}$ are the direct power and power due to reflections, and $P_{r}$ is the received power. The delay in RMS spread is

$$D_{RMS}=\sqrt{\mu^{2}-{\left(\mu \right)}^{2}}.$$

The maximum acceptable bit rate $R_{b}$ transmitted is less than or equal to $\frac{1}{10D_{RMS}}$. The AUC using the $D_{RMS}$ value for variation in LEDs is shown in Table 2. When the number of LEDs is reduced from 4 to 2, the AUC increases by 9%; that is,

$$\frac{1.7951-1.2573}{1.795}\times 100.$$

This measure of $D_{RMS}$ provides an upper bound for the data rates, and a reduction in LEDs improves the data rate or the light rays received at the same time.
